# Changes in school environment, awareness and actions regarding overweight prevention among Dutch secondary schools between 2006–2007 and 2010–2011

**DOI:** 10.1186/1471-2458-13-672

**Published:** 2013-07-19

**Authors:** Saskia W van den Berg, Jochen Mikolajczak, Wanda JE Bemelmans

**Affiliations:** 1 Centre for Nutrition, Prevention and Health Services, National Institute for Public Health and the Environment (RIVM), P.O. Box 1, 3720, BA Bilthoven, The Netherlands

**Keywords:** Overweight prevention, Secondary schools, Obesogenic environment, Awareness, Policy, Longitudinal

## Abstract

**Background:**

Schools can be an important setting for the prevention of overweight. This nation-wide survey investigated changes in the obesogenity of the school environment, the awareness of schools regarding overweight, school health policy, and actions taken by schools to prevent overweight.

**Methods:**

In 2006/2007 and 2010/2011, questionnaires were sent to all Dutch secondary schools, (n = 1250 and n = 1145, response rate 44% and 33% respectively, repeated data for 187 schools).

**Results:**

The percentage of schools with vending machines for soft drinks (~90%) and sweets (~80%) remained fairly stable, whereas slightly more schools indicated to have a canteen (87%-91%). The food supply was reported to be healthier in 2010/2011 compared to 2006/2007. Canteens and/or vending machines offered more often fresh fruits (+8%), sandwiches (+11%), water (+11%) and salad (+7%) and less often sugar sweetened soft drinks (−10%). However, unfavorable changes such as an increase in the supply of pizza slices (+13%) and milk and yoghurt drinks with added sugar (+12%) were also reported. Between 2006/2007 and 2010/2011, the presence of water coolers increased (12% versus 33%) as well as facilities for physical activity (67% versus 77%). However, more schools had vending places of unhealthy foods in the vicinity (73% versus 85%). Compared to 2006/2007, a higher percentage of schools indicated that they have taken actions to stimulate healthy eating behavior (72% versus 80%) or to prevent overweight (34% versus 52%) in 2010/2011. Less schools indicated that they expect to pay more attention to overweight prevention in the near future (56% versus 43%), but none of them expected to pay less attention.

**Conclusions:**

Several aspects of the school environment changed in a positive way. However, schools should be encouraged to contribute to the prevention of overweight, or to continue to do so.

## Background

Worldwide, obesity remains one of the major public health issues [1]. In the past decades, overweight and obesity prevalence has substantially increased, also in the Netherlands. In 2009, 12.9% of the boys and 14.8% of the girls in the Netherlands aged 2–21 years were overweight and respectively 1.8% and 2.2% were obese [2]. This is a two to three fold increase of the 1980 overweight prevalence rates and a four to six times increase in the 1980 obesity prevalence [2].

Prevention is considered to be an appropriate way to counteract overweight among adolescents, though this is a major challenge [3]. An important aspect of overweight prevention is to tackle the obesogenic environment [4]. The latter refers to an environment that promotes an unhealthy diet and physical inactivity and thereby contributes to the development of an excessive fat mass.

Since schools reach many children, and children spend a lot of time at school, schools are considered to be a relevant and important setting for the prevention of overweight and obesity [5]. Several cross-sectional studies investigated school food environments and policies [6-10] and their association with children’s dietary intake [11-13] and body weight [14-16]. However, longitudinal studies conducted on this topic are limited [17].

During the 2006–2007 school year, we conducted a nation-wide written survey on school environment, school policy, awareness of the schools regarding overweight and actions taken to prevent overweight at Dutch secondary schools [10]. The main findings were that unhealthy drinks and foods were widely available at secondary schools. In addition, one third of the schools indicated that overweight had increased among students and half of the schools considered themselves to be (co)responsible for the prevention of overweight. Only three percent of the schools had a policy on the prevention of overweight.

Since the 2006–2007 school year several developments in national policies took place regarding the prevention of overweight and obesity. In 2009, the Dutch Government launched a policy document on overweight. In addition, national policies were intensified through several initiatives as amongst others the Dutch Covenant on Healthy Weight [18]. This covenant is a collaboration of 27 actors from national and local governments, industry and civil society organizations who collectively committed to fight against the rising trend of overweight and obesity. A concrete example of an initiative from this covenant is promoting the so called JOGG approach (which is based on the successful French EPODE methodology [19]) to prevent overweight among young persons (See for more detailed information: http://www.epode-international-network.com/programmes/jogg). Another concrete example is the sub covenant ‘school’ that includes the The Healthy School Canteen programme from the Dutch Nutrition Centre [20]. The goal set by the Dutch Ministry of Public Health, Welfare and Sports of realizing 100% health school canteens by 2015 has been adopted within this sub covenant. In 2009, also a policy document on Sports, Physical Activity and Education was launched in which local authorities and organizations acting in the field of education and sports collaborate. Main aim of this policy was to achieve that 50% of the children aged 4–17 satisfy the physical activity norm in 2012. Another relevant development since 2006–2007, is the establishment of the RIVM Centre for Healthy Living, which supports the delivery of efficient and effective local health promotion in the Netherlands for different settings including school.

Of interest is whether these developments in recent years have led to improvements in the school environ-ment. The above mentioned survey was repeated during the 2010–2011 school year, yielding the opportunity to investigate whether improvements have occurred and allows us to assess the magnitude and the direction of the potential changes therein. Aim of the current study was to investigate changes over time in the school environment, the awareness of schools regarding overweight and changes in school policy and specific actions taken to prevent overweight.

## Methods

### Study design

In 2006–2007 a first national survey on nutritional and physical environment at Dutch secondary schools was conducted (in this paper referred to as ‘baseline’). In 2010–2011, a second national survey was carried out (in this paper referred to as ‘follow-up). Both surveys were conducted through a postal questionnaire and consisted of two mailings. The first mailing was sent to all secondary schools of the Netherlands in November 2006 (baseline) and January 2011 (follow-up) and a second mailing was performed in January 2007 (baseline) and March 2011 (follow-up) to schools that had not responded so far to increase the total response. Together with the second mailing a non-response card was sent to the schools in which the reason for non-response and some main questions from the original questionnaire (selected questions differed between baseline and follow-up) were queried.

In the Netherlands, children who attend a secondary are aged 12 to 18. In addition, the majority of the secondary schools consist of different sites. To increase the readability of this paper ‘school sites’ will be referred to as ‘schools’.

The design and the response rate (44%) of the baseline survey have been extensively described by Scholtens et al. [10]. At follow up, 375 of the 1145 approached schools completed the questionnaire, from which 202 responded on the first mailing and 173 on the second. The non-response card was returned by 117 schools. Main reason for non-response was the fact that schools are confronted with many requests for participation in a study (50% of the schools indicated that this was a reason for non-response). For this study, no ethical approval was necessary according to the Dutch Central Committee on Research involving Human Subjects http://www.ccmo.nl because the questionnaires were not directed at children, no direct health related questions had to be answered and no medical investigations were included.

### Study population

This study included schools that filled out the questionnaire at both baseline and follow-up (n = 187). The 187 schools included in this study did not differ on school size, school level (vocational, mixed or higher) and degree of urbanization compared to all 577 and 375 schools (including the 187 schools with repeated measurements) and compared to the 390 and 188 schools (excluding the 187 schools with repeated measurements) that completed the questionnaire at respectively baseline and follow-up.

### Questionnaire

The baseline questionnaire consisted of 80 questions [10]. Most questions were repeated in the follow-up questionnaire. In addition, several questions were added to the follow-up questionnaire which finally consisted of 102 questions. The baseline and follow-up questionnaire were both divided in six parts, 1) general characteristics of the schools; 2) the school environment (including questions on the canteen, water coolers and vending machines for drinks, candy and fresh fruit); 3) health education; 4) participation in projects on overweight prevention; 5) school’s policy on health, diet, physical activity and overweight; 6) closing questions (e.g. who completed the questionnaire). Questions with regard to health education and participation in projects on overweight prevention were not analyzed in this study, because within a school level the curriculum is fairly similar between schools and the way of inquiring differed between baseline and follow-up.

#### School environment

For the canteen 11 specific types of ‘healthy’ and 11 types of ‘less healthy’ foods were queried and for the drinks vending machines this was done for 4 types of ‘healthy’ and 4 types of ‘less healthy’ drinks through pre-coded questions. Respondents could mark whether a particular food item was sold at their school. In addition, school representatives were asked to value the proportion of unhealthy and healthy foods in canteen or vending machines. For example “How would you describe the proportion of high caloric and low caloric drinks in soft drink vending machines? The five predefined answer options were: 1) nearly all high caloric drinks; 2) more high caloric than low caloric/healthy drinks; 3) equal amount of high caloric and low caloric/healthy drinks; 4) more low caloric/healthy than high caloric drinks; and 5) nearly all low caloric/healthy drinks”.

#### Awareness and responsibility of the schools towards overweight and school policy

School representatives where asked to indicate whether they observe an increase in the number of students at their school (yes; no; I do not know) and whether they think that overweight is more prevalent at their school compared to the general population of children aged 12–18 (yes; no; just as much). In addition, they were queried who they held responsible for the prevention of overweight among their students (school; parents; students themselves; government; no opinion; otherwise, namely…. (possible to indicate more answers)) and whether they expect that their school will be pay more attention to overweight in the future (yes, more attention; no, just as much attention; no, less attention).

Furthermore, it was asked whether the school has a policy on general health, (healthy) nutrition, sports and physical activity, and overweight. For example “Does your school have a policy on (healthy) nutrition? The five answer options were: 1) yes, lay down in writing and part of the general health policy, 2) yes, lay down in writing, but no part of the general health policy, 3) yes, verbally expressed, 4) no, and 5) I do not know.” In addition, the questionnaire contained questions about the compliance to those policies and about obstacles/barriers that have been experienced by the implementation of those policies. For example, “Do you think that the policy on nutrition, physical activity and/or overweight are well complied to? Answer options were 1) very well; 2) well; 3) sufficient; 4) insufficient; 5) poor; 6) not applicable.”

#### Actions to prevent overweight

School representatives were asked to indicate whether several predefined general measures are taken by the school to stimulate healthy eating behavior (n = 7), to discourage unhealthy eating behavior (n = 9), to stimulate physical activity (n = 7), and regarding overweight (n = 3),

For this study, the questions relevant for the study question that were inquired (in a similar way) in both questionnaires were selected. This resulted in 18 questions describing the school environment (including all questions on food supply) and 14 questions on school policy (including awareness of schools and specific actions undertaken by schools to prevent overweight). In some cases, questions were combined to one outcome variable or were split into several outcome variables.

### Data analysis

In total, 58 outcome variables were a priori chosen to be tested. In addition, specific actions undertaken by schools to prevent overweight were also evaluated but not tested. Changes in dichotomous outcome variables were analyzed using conditional logistic regression. Outcome variables were added as dependent variable and the point of measurement as independent variable (0 ‘baseline’, 1 ‘follow-up). School was added as stratum to the regression models to ensure comparison within schools. All models were adjusted for (changes in) school level and school size. Changes in ordinal outcome variables were analyzed by linear regression analysis. We tested whether the change over time in the outcome of interest (added as dependent variable) differs statistically significant from zero by evaluating the intercept of this model. Potential confounding variables (school level and size) were added as centered variables (deviation from the mean) to the models to maintain the possibility to interpret the intercept. At baseline, five outcome variables (i.e. content of soft drink vending machines, sweets/candy bars vending machines and the canteens, schools opinion about the prevalence of overweight among the students compared to the general population, and the action “it is forbidden to sell certain unhealthy foods at school”) differed between school levels [10]. Therefore, interaction between changes in those outcome variables and school level was investigated, which however did not appear to be the case. To gain more insight in the robustness of our findings, a non-response analysis was performed, with a focus on actions undertaken by schools regarding the prevention of overweight as this was queried in the non-response cards distributed at baseline as well as the follow up. P-values below 0.05 were considered to be statistically significant. Data analysis was conducted using SAS software version 9.2 (SAS Institute, Inc., Cary, NC, USA).

## Results

### School characteristics

Main characteristics of the schools at baseline and follow up are presented in Table 1. Of the 187 schools, 58% (n = 108) was located in a rural area, whereas 42% (n = 78) was located in urban area. Most of the schools (92%) reported the same educational level at baseline and follow up. Approximately, 44% of the schools were vocational education schools, 44% were mixed schools and 12% were higher education schools. The median school size was about 650 students at baseline and follow up. Most questionnaires were completed by more than one person (≈55%). More than 70% was (co-) completed by the principal or (assistant) manager of the school, approximately half of the questionnaires was (co-) completed by a teacher in biology, in physical activity, or in health and hygiene, and a quarter was (co-) completed by an employee of the canteen at school at baseline as well as at follow up.

**Table 1:** **Content of the questionnaire and main characteristics of the secondary schools at baseline and follow up** (**n** = **187**)

	**Baseline**	**Follow up**
	**2006-2007**	**2010-2011**
*Content of the questionnaire*		
**Number of questions on:**		
The school environment	28	32
School policy	17	21
*Characteristics*		
**Degree of urbanisation (%)**		
Rural	58	58
Urban	42	42
**School size**^**1,2 **^**(median)**	**670**	**654**
< 500 (%)	37	41
500-1000(%)	29	25
>1000(%)	33	34
**Educational level**^**3 **^**(%)**		
Vocational	44	44
Mixed	45	44
Higher	10	12
**Who (co-) completed the questionnaire? (%)**		
Prinicipal or (assistent) manager	76	72
Teacher^4^	45	54
Employee of the canteen	24	23

^1^ 36 schools (19%) moved from school size category between baseline and follow up.

^2^ Vocational education schools included schools only offering ‘preparatory vocational education’. Mixed schools included schools offering ‘preparatory vocational education’ senior general education’ and/or ‘university preparatory education’. Higher education schools included schools offering ‘senior general education’ and or ‘university preparatory education’,

^3^ Of 16 schools (8.5%) the educational level changed between baseline and follow up.

^4^ Teacher in biology, in physical activity or in health and hygiene.

### School environment

At the majority of the schools a soft drink vending machine, a vending machine containing sweets and candy bars and/or a canteen was present (Table 2). There was no difference between baseline and follow up regarding the presence and the number of vending machines present at schools, whereas the number of schools with a canteen was slightly higher at follow up (p = 0.03). The percentages of schools who indicated that their vending machines and/or canteen contained a less favorable selection of food and drinks was statistically significant lower in 2010–2011 compared to 2006–2007. Compared to baseline, school representatives indicated that their soft drink vending machines contained more often water (60% vs 71%), but also milk and yoghurt drinks with added sugar (16 vs 28%) at follow up. Canteens at schools offered more often fresh fruit (26% vs 34%), sandwiches (56% vs 67%) and salad (6% vs 13), but also pizza slices (17% vs 30%) at follow up compared to baseline. A total of 95% of the schools indicated that they offered sugar sweetened soft drinks in canteens and/or vending machines at baseline, whereas this percentages was 10% lower at follow up. At baseline as well as at follow up 83% of the schools indicate that school management can influence the food supply offered in their canteen.

**Table 2:** The school environment

**Outcome variable**^**1**^	**Baseline**	**Follow up**	**P-value**^**2**^
	**(2006/2007)**	**(2010/2011)**	
	**% (n)**	**% (n)**^**1**^	
**Soft drink vending machine present at school**	**91 (170)**	**89 (167)**	**0.59**
**Soft drink vending machines contain:**			**0.02**
More unhealthy than healthy drinks	53 (83)	45 (71)	
Unhealthy and healthy drinks equally.	38 (60)	33 (52)	
Less unhealthy than healthy drinks	9 (14)	22 (34)	
**Vending machine present at school that contains sweets /candy bars**	**81 (151)**	**79 (146)**	**0.63**
**Sweets/candy bars vending machines contain:**			**0.04**
Mainly unhealthy foods	64 (80)	55 (69)	
A good balance between unhealthy and healthy foods	37 (46)	41 (52)	
Mainly healthy foods	0 (0)	4 (5)	
**Water cooler present at schools**	**12 (22)**	**33 (60)**	**<0.0001**
**Vending machines present at school that contains fresh foods**	**11 (19)**	**11 (19)**	**0.91**
**Canteen present at school**	**87 (161)**	**91 (170)**	**0.03**
**Proportion of healthy and unhealthy foods present in the canteen:**			**0.004**
Mainly less healthy foods	14 (21)	9 (14)	
More less healthy than healthy foods	34 (51)	27 (40)	
Equal distribution of healthy and less healthy foods	31 (46)	28 (42)	
More healthy than less healthy foods	14 (21)	28 (42)	
Mainly healthy foods	8 (12)	9 (13)	
**Availability of specific healthy foods and/or drinks**^**4**^			
Drinking water offered in canteen and/or vending machine	60 (109)	71 (129)	0.03
Fresh fruits offered in canteens	26 (48)	34 (63)	0.03
Sandwiches offered in canteens	56 (104)	67 (124)	0.003
Salad offered in canteens	6 (10)	13 (21)	0.06
**Availability of specific less healthy foods and**/**or drinks**^**4**^			
Sugar sweetened soft drinks offered in canteen and/or vending machine	95 (174)	85 (156)	0.003
Milk and yoghurt drinks with added sugar offered in vending machines	16 (29)	28 (51)	0.003
Pizza slices offered in canteens	17 (31)	30(55)	0.0006
**There is a supermarket**, **gas station or fast food restaurant in the neighborhoods of the school.**	**73 (127)**	**85 (148)**	**0.003**
**There are facilities at and around the school property where the student can be physically active.**	**67 (120)**	**77 (138)**	**0.04**
**Indicated percentage of students who walk to school or travel by bike (*median*** [*Q1*;*Q3*])^*3*^	**80 [70;90]**	**80 [70;90]**	**0.31**
**There is sufficient space for students to park their bikes at bike parks**	**85 (159)**	**95 (173)**	**0.01**

^1^% yes (n) or otherwise indicated.

^2^ P for change. Changes in dichotomous outcome variables are tested using conditional logistic regression. Changes in ordinal outcome variables are analyzed by linear regression analysis. All models are adjusted for (changes in) school level and school size. P-values below 0.05 are considered to be statistically significant.

^3^ Q1:first quartile, Q3: third quartile.

^4^ It was also queried whether other ‘less healthy foods’ like: sport drinks, fruit juices with added sugar, candy bars, sweets, cakes, fried snacks, Russian salad, ice creams crisps and other ‘healthy foods’ like: (fresh) fruit juices without added sugar, artificially sweetened soft drinks, rice cakes, soup, salad,yoghurt were offered in canteens or vending machines but no statistically significant change was observed.

The presence of water coolers at schools was higher in 2010–2011 than in 2006–2007 (p < 0.0001). At follow up, 40% of the schools indicated to provide water for free, which was 14% at baseline. At the majority of the schools (85%) where water was not freely distributed, the price of one cup is ten eurocent.

Compared to baseline, the percentage of schools that had a supermarket, fast food restaurant or gas station in the neighborhood (within 1 km of the school) was statistically significant higher at follow up (73% versus 85%). At follow up, 77% of the schools indicated to have facilities at or around the school property where students can be physically active, compared to 67% of the schools at baseline. This increase was mainly due to soccer fields on the school property.

### Awareness and responsibility of the schools towards overweight and school policy

The percentage of schools that indicated that overweight was not more prevalent among the students of their school than among the 12 to 18 year old children in the general population was lower at follow up compared to baseline (p 0.04; Table 3). There was no statistically significant difference between baseline and follow up in the opinion regarding the responsibility for the development of overweight among students (about 40% of the schools found themselves responsible) and whether the prevalence of obesity has increased among their students.

**Table 3:** Awareness and responsibility of the schools towards the overweight problem and school policy

**Outcome variable**^**1**^	**Baseline**	**Follow up**	**P-value**^**2**^
	**(2006/2007)**	**(2010/2011)**	
	**% (n)**	**% (n)**	
**The prevalence of obesity has increased among students**			**0.27**
No	40 (72)	40 (72)	
Yes	34 (61)	29 (52)	
Don’t know	27 (48)	31 (55)	
**Overweight is more prevalent among the students at school than in the general population**			**0.04**
Yes	6 (10)	8 (13)	
Equal	12 (21)	20 (34)	
No	82 (140)	73 (124)	
**Responsibility for the prevention of overweight among students**			
Schools	40 (72)	37 (67)	0.38
Parents	98 (177)	98 (177)	1.00
Students	81 (146)	77 (139)	0.17
Government	15 (27)	17 (32)	0.49
*Policy*			
**School has policy on:**			
General health	12 (22)	17 (30)	0.11
(Healthy) nutrition	49 (85)	57 (96)	0.16
Physical activity	55 (86)	52 (82)	0.58
Overweight	11 (19)	17 (28)	0.14
**Compliance to policy on diet, physical and/or overweight at schools?**^**3**^			**0.76**
Very well	0 (0)	4 (2)	
Well	32 (17)	26 (14)	
Sufficient	59 (31)	64 (34)	
Insufficient	9 (5)	6 (3)	
Poor	0 (0)	0 (0)	
**School experiences barriers/obstacles by implementation of their policy on diet, physical and**/**or overweight**^**3**^	**63 (40)**	**58 (43)**	**0.47**

^1^% yes (n) or otherwise indicated.

^2^ P for change. Changes in dichotomous outcome variables are tested using conditional logistic regression. Changes in ordinal outcome variables are analyzed by linear regression analysis. All models are adjusted for (changes in) school level and school size. P-values below 0.05 are considered to be statistically significant.

^3^ Among schools with a policy on diet, physical and/or overweight at baseline and follow up (n = 97).

The proportion of schools with a policy on (healthy) nutrition (49% versus 57%) or a policy on overweight prevention (11% versus 17%) seemed to be higher at follow up than at baseline, however differences were not statistically significant. At baseline and follow-up, approximately 90% of the schools evaluate the compliance to those policies as sufficient. However, about 60% of the schools with a policy on diet, physical and/or overweight at baseline and follow-up experience barriers (mainly lack of time and no financial resources) that hinder the implementation of those policies.

### Actions taken to prevent overweight

Compared with baseline, a higher percentage of schools indicated that they have taken actions to stimulate healthy eating behavior (p = 0.08) or actions to prevent overweight (0.0009) at follow up (Table 4). With regard to specific actions, 46% attempted to offer a good balance in food and beverages, 20% participated in the national project “Healthy School Canteen Programme”, 56% has banned the sale of certain unhealthy foods in the canteen and 18% are tackling the parents about the eating behavior of their child at the follow up measurement. Those percentages were 9% lower at baseline. There was no change between baseline and follow up in the percentage of schools that indicated to have taken actions to stimulate physical activity. A statistically significant lower percentage of schools indicated that they expect to pay more attention to overweight prevention in the future (56% versus 43%), but none of them expected to pay less attention.

**Table 4:** Actions taken by the schools to prevent overweight

**Outcome variable**^**1**^	**Baseline**	**Follow up**	**P-value**^**2**^
	**(2006/2007)**	**(2010/2011)**	
	**% (n)**	**% (n)**	
**Actions taken to stimulate healthy eating behavior:**	**72 (127)**	**80 (140)**	**0.08**
Healthy products are made less expensive than unhealthy products	26 (46)	30 (52)	
Introduction of water coolers	15 (27)	26 (45)	
Participation in national project “Healthy School Canteen Programme”	11 (19)	20 (35)	
Canteen offers wide variety of healthy foods	28 (49)	30 (52)	
Vending machines offers wide variety of healthy foods	17 (29)	19 (34)	
Attempt to offer a good balance in food and beverages	34 (60)	46 (80)	
After-school meetings organized on healthy diet	7 (13)	12 (22)	
Other	16 (27)	20 (35)	
**Actions taken to discourage unhealthy eating behavior:**	**89 (155)**	**93 (161)**	**0.23**
It is forbidden to sell certain unhealthy foods in the canteen	38 (65)	56 (98)	
Parents are tackled about the eating behavior of their child	9 (16)	18 (32)	
It is forbidden to eat in the classroom	79 (137)	87 (151)	
Adding healthy products to vending machines	43 (75)	44 (76)	
Regulation of media that stimulate less healthy eating behavior	4 (7)	1 (1)	
Other	26 (45)	35 (60)	
**Actions taken to stimulate physical activity:**	**76 (135)**	**80 (142)**	**0.21**
School stimulates the students to be physically active during breaks	20 (36)	17 (31)	
Collaboration with sport associations	32 (57)	26 (46)	
School often organizes activities for the students to be physical active after school hours.	62 (110)	57 (102)	
School policy on physical activity active after school hours.	17 (31)	12 (21)	
Other	8 (14)	26 (47)^3^	
**Actions taken to prevent overweight:**	**34 (49)**	**52 (74)**	**0.0009**
After-school meetings organized on overweight	5 (7)	5 (7)	
There are guidelines to identify ant to help students with overweight	15 (21)	14 (20)	
Students who are overweight get more attention during physical activity classes	10 (14)	12 (17)	
Other	13 (19)	49 (70)^4^	
**Regarding overweight in the future**, **school expects to pay**			**0.03**
More attention	56 (98)	43 (76)	
Equal attention	44 (77)	57 (100)	
Less attention	1 (1)	0 (0)	

^1^% yes (n) or otherwise indicated.

^2^ P for change. Changes in dichotomous outcome variables are tested using conditional logistic regression. Changes in ordinal outcome variables are analyzed by linear regression analysis. All models are adjusted for (changes in) school level and school size. To reduce the number of tests performed and the chance of false positive findings, it was a priori decided to only evaluate, but not test changes in specific actions undertaken by schools. P-values below 0.05 are considered to be statistically significant.

^3^ Includes 36 schools (20%) that indicated to have discussion with the local government about facilities to be physical active. This action is only queried at follow-up and not at baseline.

^4^ Includes 61 schools (43%) that indicated that they bring students with overweight and their parents in contact with (health) professionals. This action is only queried at follow-up and not at baseline.

## Discussion

The results of these longitudinal analyses suggested that the environment inside and also partly outside secondary schools has become less obesogenic in 2010–2011 compared to 2006–2007. Schools reported that the supply of foods and drinks in vending machines and canteens has become healthier. This favorable change was illustrated by a decreased availability of sugar sweetened soft drinks and an increased availability of drinking water, fresh fruit, salad and sandwiches at secondary schools. However, unfavorable changes were also observed, as an increased availability of pizza slices and milk and yoghurt drinks with added sugar. With regard to physical activity, there was an increase in the presence of soccer fields at schools, and also the environment outside the school improved with respect to the facilities available for students to be physical active. However, there are also more facilities in the neighborhood of the schools for students to buy less healthy foods. More schools indicated that they have taken actions to stimulate healthy eating behavior or actions to prevent overweight. A lower percentage of school representatives indicated that they expect to pay more attention to overweight prevention, but none of them expected to pay less attention to the issue of overweight in the near future.

One of the strengths of our study is its longitudinal design. This allowed us to include a relatively large group of secondary schools in the analyses, despite the fact that overall response rates dropped between baseline (school year 2006–2007) and follow-up (school year 2010–2011). This may have led to selection bias, for example if the response was higher for school representatives with a higher interest in the prevention of overweight. Non-response analysis, including data obtained by non-response cards, indeed showed that the prevalence of schools that had taken actions to prevent overweight was lower among non-responders compared to responders, at baseline as well as follow-up. However changes over time, which is the most important outcome indicator in this manuscript, did not differ between responders and non-responders (only possible for the schools that filled out a non-response card at baseline as well as at follow-up). In addition, school level, size and location did not differ between schools that completed both questionnaires with those that completed only one of them. Finally, our results (based on 187 schools with repeated measurements) are confirmed by the results of a cross-sectional comparison between the 375 schools that filled out the questionnaire at follow-up and the 515 schools included at baseline (results not part of this paper) [21]. So, overall we feel confident about representativeness of the reported changes over time, despite disappointing response rates.

In our study, we only examined changes in self reported indicators of the obesogenic environment at Dutch secondary schools. This may be subject to recall bias and/or social desirability bias. The reported favorable changes in food supply are reflected to some extent in the reported changes in the availability of specific healthy and less healthy products in the canteen and/or vending machines at schools (eg. increase in fresh fruits and sandwiches and a decrease in sugar sweetened soft drinks). However, less favorable trends were also observed (eg. increase in pizza slices and milk and yoghurt drinks with added sugar). More objective exposure data could be collected by visiting schools and/or using purchase data.

In this study, data on body mass index and dietary behavior of students was not available. Such measurements would provide more objective outcome measures of the potential impact of the changes in the obesogenic environment of Dutch secondary schools. Literature shows for example that attending a middle or high school without stores or snack bars was associated with a reduced sugar-sweetened beverage consumption in children [11]. In addition, consumption of sugar-sweetened beverages has been linked with an increased risk to develop overweight among children [22]. There is also evidence for a direct association between food supply at schools and risk of obesity among children. For example, a cross-sectional research among middle school children showed that the availability of unhealthy foods and drinks in vending machines was associated with a higher BMI among middle school children [15]. On the other hand, the availability of such foods in the cafeteria was associated with a lower BMI. In addition, none of these associations was found for high school students. As far as we know, one longitudinal study has been performed on this topic, which did not find an association between the sale of unhealthy foods and drinks and weight gain among middle school children [17]. More longitudinal research among middle as well as high school children is needed to elucidate this topic.

In contrary to many countries, the Netherlands does not have a tradition of providing meals at school. Most students bring their packed lunch from home, and –either in addition or instead- they can use the opportunity to buy foods and drinks at school. Thus the impact on food supply at schools on the dietary pattern of students may be less than in countries providing school meals.

For the interpretation of our results it should be kept in mind that multiple tests (n = 58) were performed. By using a p-value below 0.05 as a threshold for statistically significance (58*0.05), three false positives findings can be expected [23]. As this study showed statistically significant changes in 17outcome variables, the majority of our findings will be true positive findings.

Obesity policies in the Netherlands do not involve obligatory regulations or legislation, but is rather dependent on self-regulation, the mobilization of action, projects and campaigns e.g. by national and local health promoting institutes. In recent years, developments in those national policies have been started or continued in the Netherlands (for examples see introduction). In addition, financial resources for projects that aimed to tackle overweight and obesity in the Netherlands were also increased. It is tempting to assume that the observed improvements in the obesogeneity of the environment at secondary schools are, at least in part, the results of those developments.

## Conclusions

Our longitudinal analyses indicate some positive changes in the obesogenic environment at Dutch secondary schools. For example, about 25 000 more students now have access to water coolers (38 schools indicated that they have introduced a water cooler; median school size at follow-up 654), so for these students a healthy alternative has at least become available. However our results also show that there is room for further improvement with respect to the obesogenic environment, the aware-ness of schools, and the school policy regarding overweight. In line with recommendations of the European Commission and the WHO [24,25], schools should remain a priority setting for the prevention of overweight and obesity in national and international policies on health and education. Taking specific measures or actions that contribute to the reduction of the obesogenic environment at schools should remain an important focus of their preventive efforts.

## Competing interests

The authors declare that they have no competing interest.

## Authors’ contributions

All authors commented critically on the data analysis, interpretation of the results and manuscript drafts and agreed on the final version. SB performed the statistical analysis and drafted the manuscript. JM designed and coordinated the second survey and collected the follow-up data. WB originated and supervised the whole study. All authors read and approved the final manuscript.

## Pre-publication history

The pre-publication history for this paper can be accessed here:

http://www.biomedcentral.com/1471-2458/13/672/prepub
